# Outcomes of Laparoscopic Gastric Greater Curvature Plication in Morbidly Obese Patients

**DOI:** 10.1155/2017/7989714

**Published:** 2017-08-16

**Authors:** N. Khidir, M. Al Dhaheri, W. El Ansari, M. Al Kuwari, D. Sargsyan, M. Bashah

**Affiliations:** ^1^Department of Metabolic & Bariatric Surgery, Hamad General Hospital, Doha, Qatar; ^2^Department of Surgery, Hamad General Hospital, Doha, Qatar; ^3^College of Medicine, Qatar University, Doha, Qatar; ^4^School of Health and Education, University of Skövde, Skövde, Sweden; ^5^Weill Cornell Medicine-Qatar, Doha, Qatar; ^6^Qatar Metabolic Institute, Doha, Qatar

## Abstract

**Background:**

Laparoscopic gastric greater curvature plication (LGGCP) is a restrictive bariatric procedure without gastrectomy. However, limited literature on effectiveness of gastric plication exists.

**Objectives:**

We assessed LGGCP's efficacy, effects on associated comorbidities, safety and the rate of complications, and patient satisfaction with LGGCP's outcomes among morbidly obese patients.

**Method:**

Analysis of retrospectively data collected from medical records of 26 patients who had undergone LGGCP at Hamad General Hospital, Qatar, during 2011-2012.

**Results:**

Most patients (92%) were Qatari nationals. The sample's mean age was 35.1 years. Mean duration of hospital stay was 3.9 ± 1.2 days. Mean preoperative BMI was 40.7 kg/m^2^ that decreased at 2 years to 34.6 kg/m^2^. LGGCP's effects on comorbidities were such that 7.6% of patients experienced resolutions of their comorbidities. There were no mortality or postoperative complications that required reoperation. Six patients (23%) were satisfied with the LGGCP's outcomes while 10 patients (38.5%) underwent sleeve gastrectomy subsequently.

**Conclusion:**

LGGCP had acceptable short term weight loss results, exhibited almost no postoperative complications, and improved patients' comorbidities. Despite the durability of the gastric fold, some patients regained weight. Future research may assess the possibility of an increase in the gastric pouch size postplication associated with weight regain.

## 1. Introduction

Qatar witnesses a substantial increase in the prevalence of obesity that is linked to the rapid socioeconomic development of the country. Given that 40% of the general population and 42% of all Qataris are obese, concerted efforts are required to solve and mitigate this important public health challenge that has serious health impacts [1, 2]. Preventive measures to combat such obesity trend have included general public health campaigns, encouragement of physical activity, and nutrition awareness programs that advocate low calorie diets. In addition, for morbid obesity that is resistant to lifestyle modification and medical treatment, bariatric surgery has been employed as part of the battle against obesity. Gastric volume restriction is an effective method in achieving weight loss by restricting the food intake [3].

Several restrictive and malabsorptive bariatric procedures have been implemented in Qatar. Laparoscopic gastric plication (LGP), a technique that is in the investigational stage, is a restrictive procedure that causes mechanical restriction of food intake by decreasing the stomach volume without resection [4]. In laparoscopic gastric greater curvature plication (LGGCP), a gastric tube is formed by folding the greater curvature of the stomach. However, as LGGCP remains investigational, there is dearth of local, regional, and international data on its efficacy and safety, the short term durability of the weight loss associated with LGGCP, and its effects on associated comorbidities. A recent systematic review of the published literature on LGP for the treatment of obesity yielded 14 studies [4], to conclude that additional research and long term follow-up were needed to further define LGP's role in the surgical management of obesity.

Therefore, in order to bridge this gap in the literature, the current study assessed the role of LGGCP in morbidly obese patients in Qatar in terms of its efficacy (weight loss variables; EWL% and BMI); durability of weight loss (2-year follow-up); effects on associated comorbidities (hypertension and hyperlipidemia); safety and the rate of complications that required reoperation; in addition to patient satisfaction with the outcome of LGGCP.

## 2. Method

The current study was conducted at Hamad Medical Corporation (equivalent of Ministry of Health) in Doha, Qatar. The Medical Research Centre at Hamad Medical Corporation approved the research protocol (Protocol #15117). Data was then retrospectively collected from the medical records of patients who had undergone LGGCP at HGH between 2011 and 2012 (26 patients). Most patients (92%) were Qatari nationals (8% were non-Qataris); females comprised 69% of the sample (*n* = 18), compared to 31% males (*n* = 8); and patients' mean age was 35 (±7.9) years.

All patients were seen in the clinic preoperatively. Considering patients' choice, all were offered the new investigational procedure (LGGCP) and informed of the possible benefits, risks, and potential complications, after which all agreed to sign an informed consent. As baseline routine preoperative assessment, all patients undertook upper GI endoscopy, which were all normal (insignificant findings). Patients then underwent LGGCP (February 2011–November 2012), using the same pre- and postoperative approach and surgical technique. Four patients had previous adjustable gastric banding; hence these patients had laparoscopic removal of gastric band in addition to LGGCP. Another 2 patients had symptomatic GERD (gastroesophageal reflux disease), and their intraoperative findings showed hiatal hernia; hence these patients underwent laparoscopic hiatal hernia repair in addition to LGGCP.

Our primary objective was to assess the efficacy of LGGCP and the weight loss after LGGCP. Our weight loss assessments included the postoperative change in BMI and the percentage of excess weight loss (EWL%). The study nurse measured patients' height and weight (at initial preoperative screening on the day of surgery, and at 1, 3, 6, 12, and 24 months after surgery) and calculated patients' BMI (at initial preoperative screening visit, and at 24 months after surgery). Calculation of ideal body weight was that equivalent to a BMI of 25 kg/m^2^. Assessment of the associated comorbidities and patients' satisfaction with LGGCP were both undertaken at least 24 months after surgery. There was no loss of patients for follow-up at the specified time points of measurement.

### 2.1. Surgical Procedures

**Figure 1:**
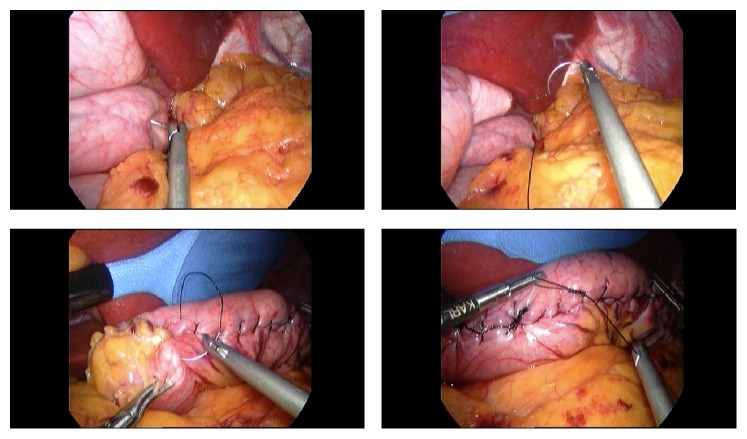
Gastric plication procedure. Suturing starts at the angle of His.

*Technique.* This was standardized. We dissected the greater omentum and short gastric vessels. The greater curvature was then folded from the angle of His up to the antrum using silk nonabsorbable sutures. Tubing of the stomach was undertaken over bougie tube size 38 F with initial two layers of continuous sutures and a second layer of interrupted sutures (Figure 1).

All patients had postoperative gastrografin meal imaging (to rule out any leaks), and the gastrografin did not show any significant stenosis or leak though irregular stomach wall due to postoperative changes and oedema. Analgesia, prokinetic antiemetic (metoclopramide), and steroids (dexamethasone) were routinely prescribed for nausea and vomiting. Oral fluid intake started 6 hours postoperatively and progressed as tolerated. All patients were seen by a bariatric dietitian and instructed to follow liquid diet for 3 weeks, after which they were then advanced to mashed and solid diet gradually.

Statistical analysis was undertaken using SPSS 20.0 (IBM Corp. Armonk, NY), with significance level set at *P* < 0.05. For descriptive analysis, we computed means and frequencies. *T*-tests compared the findings of patients who regained weight and those who did not regain weight in the postoperative period (between 1 and 2 years). Repeated weight measures over different time-points were analysed using ANOVA test.

## 3. Results

Patients' length of hospital stay ranged from 2 to 7 days (mean = 3.9 ± 1.2 days; males = 4 days, females = 3.8 days). Mean preoperative BMI was 40.7 kg/m^2^, which decreased at 2 years to 34.9 kg/m^2^. In order to assess the efficacy of LGGCP, we measured the weight variables EWL% and BMI at 1, 3, 6, 12, and 24 months postoperatively (Table 1).

**Figure 2:**
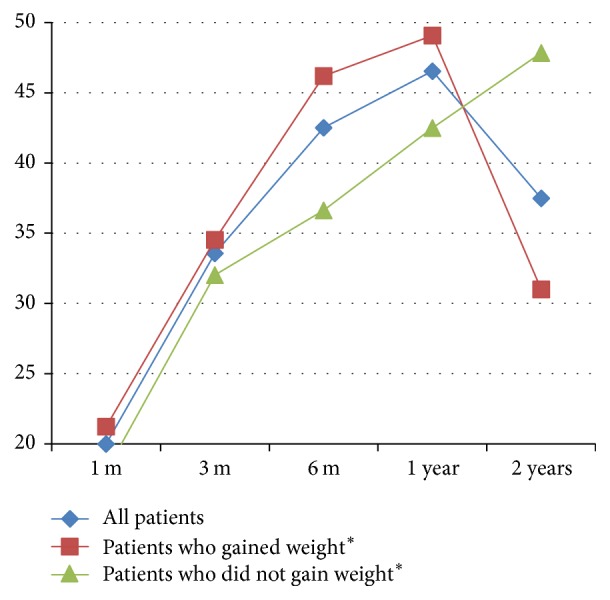
EWL% of 26 patients at 1, 3, 6, 12, and 24 months post-LGGCP. ^*∗*^Patients who gained weight = 16 and patients who did not gain weight = 9 patients.

Follow-up of EWL% at 2 years showed a loss of 37.5 ± 22.3%. At 2 years, one patient had regained weight and exceeded her initial preoperative weight. Hence the inclusion of this patient in the calculation of the sample's mean EWL% decreased the mean EWL% at 2 years from 39% (25 patients) to 37.5% (26 patients).  Figure 2 depicts the EWL% of the whole sample at 1, 3, 6, 12, and 24 months.

In terms of the associated comorbidities, preoperatively 18 patients (69%) had associated comorbidities (e.g., hypertension, hyperlipidemia, back and joint pain, hypothyroidism, end stage renal disease, and obstructive sleep apnea), whereas 8 patients (30.8%) had no comorbidities. Among those with comorbidities, measured at 2 years postoperatively, 5 patients (19.2%) improved, 2 patients (7.6%) had complete resolution of the comorbidities, and 11 patients (42.3%) showed no effect of the LGGCP on their comorbidities. Seven patients had hyperlipidemia with mean cholesterol 6.18 mmol/L that decreased to 5.33 mmol/L at 2 years.

As regards to patients' overall satisfaction with the LGGCP's short term effects, feedback from 20 patients (76.9%) indicated low satisfaction with the extent of their weight loss. Probably due to some of the dissatisfaction with their body weight after LGGCP, 10 of these patients had subsequently undertaken laparoscopic sleeve gastrectomy (LSG) at a later stage. The remaining 6 patients (23.1%) expressed high satisfaction in terms of their weight loss after LGGCP.

We did not observe any major postoperative complications nor reoperation. Only one patient (3.8%) was readmitted for nausea, vomiting, and dehydration and was treated for 3 days and discharged.

## 4. Discussion

Mechanical restriction of food intake is a main mechanism of weight loss in bariatric surgery. An earlier review of LSG as a restrictive procedure has proved its successful outcomes on both weight loss maintenance and obesity-related comorbidities [8]. However, a subsequent review [9] reported that LGP had many potential advantages when compared with LSG, as there are no anastomotic lines in LGP and hence no risk of leak from a staple line. In addition, LSG being an irreversible operation adds to its limitation as a bariatric procedure.

We are in support of the literature, where, at our Institute, we recently compared the outcomes of LGGCP (19 patients) and LSG (19 patients) [10] to find that LGGCP and LSG had almost similar weight loss at 6 months. However, we also found that the difference between preoperative and 12 months postoperative BMI was statistically different in favour of LSG. Hence for this reason, we conducted the current study of LGGCP's longer term (24 months instead of 12 months) outcomes, in order to assess the efficacy, durability of weight loss, effects on comorbidities, safety, and the rate of complications that required reoperation, in addition to patient satisfaction.

The longest follow-up after LGP (12 years) was in Iran [11], and considered EWL%< 30% during the first 6 months as failure. Using this same stringent criterion, 5 of our 26 patients (19.2%) had EWL% < 30% in the first 6 months. In addition, 16 of our patients (61.5%) regained weight by the second year of follow-up compared with their weights at one year. Though we did not routinely evaluate/follow up the gastric lumen size or assessed the gastric fold durability endoscopically, nine out of these 16 patients who regained weight had a follow-up esophagogastroduodenoscopy, that showed that 5 patients had durable gastric fold (55.6%), 4 had a partially disrupted fold (44.4%), and none had totally disrupted gastric fold. Further, we conducted a follow-up barium meal for 5 patients (those who were compliant with their follow-up appointments and presented themselves to our department), where 3 of these patients had dilated pouch, one had a disrupted fold, and 1 patient had no pouch dilatation.

**Figure 3:**
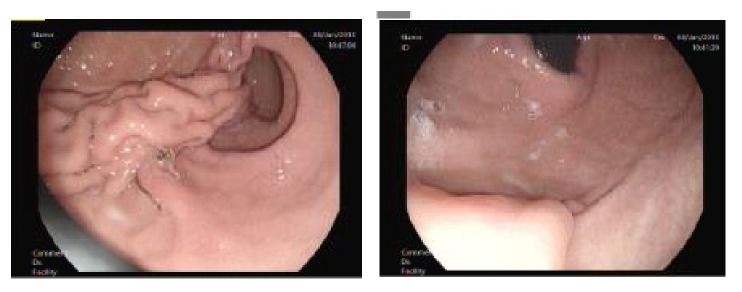
Endoscopy of female patient who regained weight 1 year after plication.

**Figure 4:**
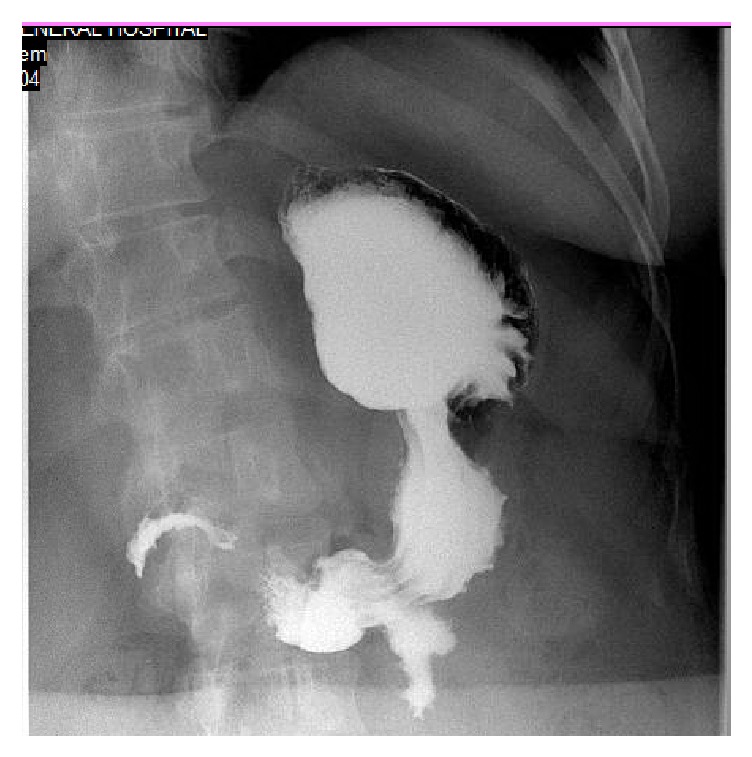
Barium meal of the same patient 4 years after surgery.

Interestingly, one of our patients reached 40.2% EWL% at 1 year, then got pregnant, and delivered. After she delivered the child, she regained weight and exceeded her initial preoperative weight. Her follow-up endoscopic evaluation showed partially disrupted fold that was also confirmed with barium meal imaging (Figures 3 and 4).

Table 2 depicts comparisons between findings of the current study and the published literature (selected variables). For instance, compared to other studies, we experienced the least EWL% at 6 and 12 months (42.5 ± 16%, 46.5 ± 20%, resp.), despite our mean preoperative BMI being somewhat similar to the mean preoperative BMI of others (e.g., [5–7]). While it is difficult to precisely speculate the reasons behind such a finding, a note to consider is that most of our patients were of Qatari nationality (i.e., not expatriates); and, globally, Qatar ranks very high in obesity, where two-thirds to three-quarters of adults are overweight or obese [12]. It could be that the eating habits of native Qataris in terms of quality and quantity (e.g., energy density foods in high quantities) are both different to other countries/cultures where other research was conducted [13]. In addition, there seems to be a different genetic profile associated with obesity among the Qatari population compared to Western populations, which could be of primary importance as the etiology of a given disease might be population-specific [12].

In connection with length of stay (Table 2), a study of 44 patients (mean BMI 38 kg/m^2^) in Canada reported a very short period of hospital stay (18 hours), while our patients exhibited a longer duration of hospital stay (mean 93.6 hours) [6]. Our observed longer LOS could be multifold, as in Qatar: (1) patients view LGGCP as a major operation, with a desire to remain longer in hospital for extra assurance; (2) staying in hospital is free of charge (no monetary costs incurred by the patient), which could lead to decreased patient's motivation to leave the hospital; and (3) patients could actually voluntarily choose to remain in hospital even after the attending physician has formally ordered a discharge (i.e., not mandatory departure).

Our complications rate compared very favourable with the literature. For instance, others reported 3 (6.8%) acute early “first week” postop complications (subphrenic abscess without gastric perforation, gastrogastric hernia, and acute respiratory distress syndrome due to severe GERD and aspiration) [6]. In contrast, we did not observe any complication (e.g., none of our patients had gastric perforation, leak, gastrogastric hernia, severe GERD or aspiration, intra-abdominal collection or abscess, and reversal of plication or experienced new onset/worsening of GERD). Even our LGGCP patients with combined hiatal hernia repair experienced absolute resolution of the GERD symptoms. We had only one patient who was readmitted because of severe nausea and vomiting on the 7th postoperative day, most likely due to postoperative edema. The patient was admitted for 3 days, treated, and then discharged.

As for LGGCP's effects on comorbidities, among our patients with comorbidities [e.g., hypertension, hyperlipidemia, back/joint pain, hypothyroidism, end stage renal disease (ESRD), and obstructive sleep apnea], at 2 years, five patients (19.2%) improved, 2 patients (7.6%) had complete resolution of comorbidities, and 11 patients (42.3%) showed no effect of LGGCP on their comorbidities. In terms of ESRD, sustained weight loss is an important first step for the management of chronic renal disease [14]. The efficacy of bariatric surgery in improving ESRD patients' renal function has been suggested [15], but due to limited research of bariatric surgery among ESRD patients, more studies are required to strengthen the evidence base. We had one male patient (53 years old) diagnosed with ESRD secondary to hypertensive nephrosclerosis and was on hemodialysis three times/week for the 7 years prior to his LGGCP. A nephrologist referred the patient to us as a kidney transplant candidate, but he had difficulty losing weight. As weight reduction is essential for the patient's renal transplant work-up and plan, we undertook LGGCP with his initial weight being 135 kg. His initial BMI (47.8 kg/m^2^) decreased to 37.9 kg/m^2^ two years after the surgery. Throughout his follow-up period, there were no significant changes in terms of his renal function parameters or frequency of dialysis, and he underwent a successful renal transplant surgery 2 years after LGGCP. Recent data available for the patient at five-year follow-up shows that he normalized his renal function parameters and is off dialysis.

We did not find research that assessed LGGCP's effects on hyperlipidemia and hence we were unable to directly compare our findings with others. Nevertheless, a systematic review of the effects of LSG on obesity associated hyperlipidemia showed that 83.5% of patients had resolution/improvement of their hyperlipidemia, and 54% experienced complete hyperlipidemia resolution [16]. We had 6 patients with hyperlipidemia, who all improved, that is, with decrease of their mean cholesterol from 6.1 mmol/L (preoperative) to 5.3 mmol/L (postoperative). Likewise, their HDL increased from a preoperative mean of 1.3 to 1.4 mmol/L, and their LDL decreased from a preoperative mean 1.4 to 1.2 mmol/L. Of the six patients, two reduced their hyperlipidemia medication dose. We found that LGGCP was not associated with as much improvement in obesity associated hyperlipidemia in comparison to LSG [16]. This calls for further research on whether the type of bariatric procedure undertaken differentially affects various hyperlipidemia parameters.

As for patient satisfaction, a nonrandomized study of LGP in Canada reported that the overall impact of weight on quality of life score (IWQOL) improved significantly at 1 year (the effect of person's weight on their self-esteem, sexual life, public distress, and work) [6]. We did not use the IWQOL, but rather we asked patients whether they were satisfied with the outcomes of their surgery or otherwise. We observed that only 6 patients (23%) were satisfied with the outcomes of their LGGCP, while the remaining 20 were unsatisfied, and 10 of our patients had a subsequent LSG at a later stage.

This study has limitations. A sample size larger than our 26 patients would have provided more information about LGGCP's efficacy and safety, as well as its effect on associated comorbidities. Moreover, as the study design was retrospective, we were unable to evaluate LGGCP's effect on diabetes mellitus and none of our 26 patients was diabetic.

## 5. Conclusion

LGGCP is a safe procedure with almost no postoperative complications. LGGCP shows acceptable short term weight loss; however patients' satisfaction seems poor. LGGCP was not associated with much improvement in patients' lipid profile. Despite the observed durability of the gastric fold, some patients regained weight. Further studies may assess the possibility of association between weight regain observed in such patients and an increase in the size of the gastric pouch.

## Figures and Tables

**Table 1:** Weight loss variables: BMI and EWL% at 1, 3, 6, 12, and 24 months postoperatively.

Variable	Preoperative	Postoperative				
		1 m	3 m	6 m	12 m	24 m
EWL%	—	20 (11.6)%	33.6 (15.5)%	42.5 (16)%	46.5 (20)%	37.5 (22.3)%
BMI	40.7	37.2 (5.6)	35.1 (5.3)	34.1 (5.2)	33.3(5.6)	34.6 (6.3)

All cell values represent mean and standard deviation; m: month; number of patients was 26 at all time points.

**Table 2:** Comparison of findings of the current study with the literature.

Study	*N*	BMI^a^ (*M*)	EWL%					LOS (M hrs)	Complication^b^
			1 m	3 m	6 m	12 m	24 m		
Current study	26	40.7	20	33.6	42.5	46.5	37.5	93.1	0
Brethauer et al. (2011) [5]	6	43.3	23.3	38.5	49.9	53.4	—	37	16.7%
Atlas et al. (2013) [6]	44	38	30.6 (40 p)	—	57 (24 p)	50.7 (13 p)	—	18	6.8%
Ramos et al. (2010) [7]	42	41	20	32 (33 p)	48 (20 p)	60 (15 p)	—	36	0

^a^Preop BMI; ^b^major postop complications; LOS: length of stay; *N*: number of patients; *M*: mean; m: month/s; hrs: hours; p: patients; —: Authors did not undertake this measurement.
